# Simultaneous Quantification of Gallic Acid, Bergenin, Epicatechin, Epicatechin Gallate, Isoquercitrin, and Quercetin-3-Rhamnoside in Rat Plasma by LC-MS/MS Method and Its Application to Pharmacokinetics after Oral Administration of* Ardisia japonica* Extract

**DOI:** 10.1155/2018/4964291

**Published:** 2018-01-14

**Authors:** Xie-an Yu, John Teye Azietaku, Jin Li, Hui Wang, Fang Zheng, Jia Hao, Yan-xu Chang

**Affiliations:** ^1^Tianjin State Key Laboratory of Modern Chinese Medicine, Tianjin University of Traditional Chinese Medicine, Tianjin 300193, China; ^2^Key Laboratory of Formula of Traditional Chinese Medicine, Tianjin University of Traditional Chinese Medicine, Ministry of Education, Tianjin 300193, China; ^3^School of Integrative Medicine, Tianjin Traditional Medical University, Tianjin 300193, China

## Abstract

*Ardisia japonica *is a well-known traditional Chinese medicinal herb used as a diuretic, for treating cough and for stopping uterine bleeding. A simple, sensitive, and reliable LC-MS/MS method was developed to determine six active compounds in rat plasma and this method was further applied to the pharmacokinetic study of these compounds after oral administration of* Ardisia japonica *extract. Acetonitrile was used to precipitate the protein in the plasma samples. Using acetonitrile and formic acid aqueous solution (0.05%) as the mobile phase, the separation of the six compounds and internal standards was achieved at a flow rate of 300 *μ*L min^−1^ on an Eclipse plus C_18_ column at an elution time of 16 min. A tandem mass spectrometer having an electrospray ionization (ESI) source was used in the detection of the analytes and internal standards using multiple reactions monitoring (MRM) in the negative ionization mode. The LLOQ was 2, 2, 4, 2, 1, and 0.4 ng mL^−1^ for gallic acid, bergenin, epicatechin, epicatechin gallate, isoquercitrin, and quercetin-3-rhamnoside, respectively. The validated method was applied to the pharmacokinetic study of gallic acid, bergenin, and quercetin-3-rhamnoside in rat plasma after oral administration of* A. japonica* extract to rats.

## 1. Introduction

*Ardisia japonica* (Thunb.) Bl, a low growing shrub belonging to the family Myrsinaceae, is one of the well-known traditional medicinal traditional herbs being probably one of the most studied among over 500 other species. It is referred to as “Ai di cha” in Chinese and is very famous for its use in treating various respiratory tract infections such as pneumonia, bronchitis, and tuberculosis. It has also been widely used in treating asthma and conjunctivitis and possesses bacteriostatic and hemostatic effects [1–3].

Some phytochemicals having been isolated and identified in* A. japonica *include coumarins, flavonoids, quinones, and saponins. Examples of these phytochemicals are 2-hydroxy-5-methoxy-pentadecenyl-benzoquione, ardisin, ardisinol I and II, bergenin, embelin, ilexol, myricitrin, quercetin, quercetrin, rapanone, and ardisianones A and B [2, 4–6]. They elicit pharmacological activities such as anti-inflammatory [4], antioxidant [7, 8], anticancer [9], anti-HIV [5, 10], and antidiabetic [6] effects. In an investigation to compare the bioactivities of six species of* Ardisia* on human hepatoma (HepG2) cells in vitro, it was observed that the chemical constituents in* A. japonica *and two other species elicited the greatest inhibitory potential against liver cancer cells. On the other hand, few methods have been developed on the pharmacokinetic of these components present in* A. japonica*.

A number of analytical methods have been previously developed in determining the chemical constituents in* A. japonica*. They include TLC-densitometric method used in determining bergenin content [11] and HPLC [12, 13]. Few analytes were determined in* A. japonica *from the methods listed above and hence may not be representative of the various types of compounds present in this herb. Besides, these methods present with long retention times and are less sensitive in the determination of the various components in this herb. Also, from our search into various literatures on this herb, no pharmacokinetic study has been conducted on this herb to the best of our knowledge.

In this study, a simple, sensitive, reliable, and validated LC-MS-MS method was presented for the simultaneous determination of six compounds in rat plasma and subsequent application to a pharmacokinetic study of these analytes after oral administration of the* A. japonica* extract to rats.

## 2. Materials and Methods

### 2.1. Materials and Reagents

**Figure 1:**
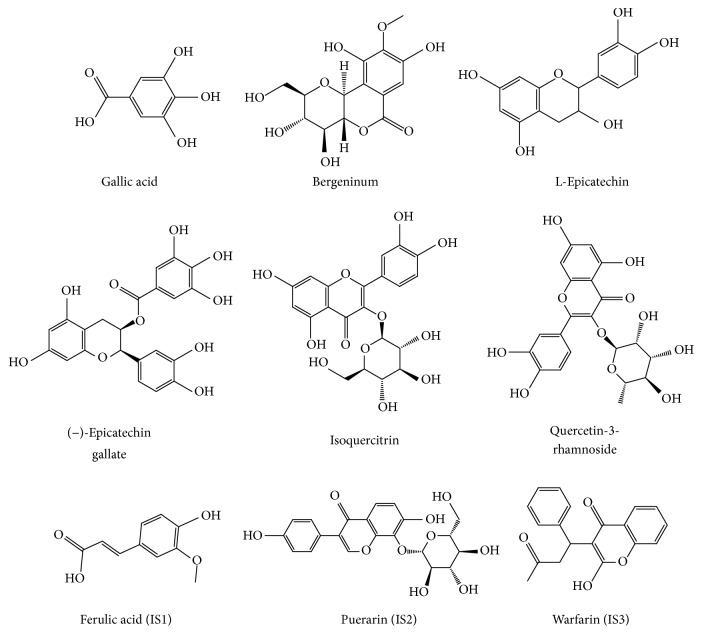
Chemical structures of 6 components and 3 ISs.

Standards including six analysis compounds (gallic acid, bergenin, epicatechin, epicatechin gallate, isoquercitrin, and quercetin-3-rhamnoside) and three internal standards (ISs) (ferulic acid, puerarin, and warfarin) (Figure 1) were purchased from National Institute for the Chinese National Institute of Control of Pharmaceutical and Biological Products (Beijing, China). Acetonitrile and methanol (Fisher, Fair Lawn, USA) and formic acid (Tedia, Fairfield, USA) were of HPLC grade. Other reagents were of analytical grade. The TCM,* A. japonica*, was offered by Anguo TCM market (Hebei, China).

### 2.2. Chromatographic and Mass Spectrometry Conditions

The LC-MS/MS system consisted of an Agilent 1200 system coupled with a binary pump (G1312A), an autosampler (G13678), a vacuum degasser (G1322A), and an API 3200 triple quadrupole mass spectrometer with an ESI source. An Eclipse plus C18 column (4.6 mm × 100 mm, 1.8 *μ*m) was maintained at 35°C and used for the separation with the mobile phase, comprising acetonitrile (A) and formic acid aqueous solution (0.05%), using a gradient elution of 25%–25% A (0–8 min), 25%–90% A (8–14 min), and 90%–95% A (14–16 min). The flow rate was set at 300 *μ*L min^−1^ and the inject volume was 10 *μ*L.

The analytes were determined in negative mode and quantified by multiple-reaction monitoring (MRM) mode. The source parameters were as follows: curtain gas, 25 psi; collision gas, 12 psi; ion spray voltage, −4500 V; temperature, 500°C; ion source gas 1, 45 psi; and ion source gas 2, 55 psi, respectively. The source parameters of every compound were optimized and are listed in Table 1. Data acquisition was operated by Analyst 1.4.2 software (AB Sciex).

### 2.3. Preparation of the* A. japonica* Extract

For the preparation of the plant extract, 1 kg* A. japonica *was extracted by refluxing with 95% ethanol for 2 h. The filtrate was concentrated using a rotary evaporator under reduced pressure. The extract yield was 8.9%.

### 2.4. Preparation of Stock Solution, Calibration Standards, and Quality Control (QC) Samples

The standard stock solutions of gallic acid, bergenin, epicatechin, epicatechin gallate, isoquercitrin, and quercetin-3-rhamnoside were prepared by dissolving each separately in methanol to achieve a concentration of 1 mg mL^−1^ for use as the stock solution. The three ISs including ferulic acid, puerarin, and warfarin were prepared using the same procedure as mentioned above with the concentration being 1 *μ*g mL^−1^. Calibration standard solutions were prepared by spiking the different concentrations of the standard mixture working solutions into plasma to achieve the concentration range of 2–500 ng mL^−1^ for gallic acid; 2–500 ng mL^−1^ for bergenin; 4–1000 ng mL^−1^ for epicatechin; 2–500 ng mL^−1^ for epicatechin gallate; 1–250 ng mL^−1^ for isoquercitrin; and 0.4–100 ng mL^−1^ for quercetin-3-rhamnoside. The QC samples were prepared by spiking 10 *μ*L of the standard stock solutions into 100 *μ*L of blank plasma to achieve LLOQ, low, medium and high concentrations of 2, 6, 20, and 200 ng mL^−1^ for gallic acid; 2, 6, 20, and 200 ng mL^−1^ for bergenin; 4, 12, 40, and 400 ng mL^−1^ for epicatechin; 2, 6, 20, and 200 ng mL^−1^ for epicatechin gallate; 1, 3, 10, and 100 ng mL^−1^ for isoquercitrin; and 0.4, 1.2, 4, and 40 ng mL^−1^ for quercetin-3-rhamnoside.

### 2.5. Preparation of Plasma Samples

100 *μ*L of plasma sample, 10 *μ*L of the IS solutions, and 10 *μ*L of 30% formic acid were added to the tube. Samples were vortex-mixed for 1 min, and 400 *μ*L of acetonitrile was then used in precipitating the protein, after which they were centrifuged for 10 min at 14,000 rpm. The supernatant was transferred into another centrifuge tube and evaporated using nitrogen gas to dryness. The dried residue was reconstituted using 100 *μ*L of methanol. The sample was further shaken and ultrasonicated for 2 min to ensure that it was well dissolved. It was subsequently centrifuged at 14,000 rpm for 10 min, and 10 *μ*L aliquot of the solution was injected into the LC-MS/MS system for analysis.

### 2.6. Method Validation

#### 2.6.1. Selectivity and the Lower Limit of Quantification (LLOQ)

The method selectivity was tested by comparing the chromatograms of six different batches of blank rat plasma samples with plasma spiked with LLOQ concentration samples of the 6 compounds and plasma samples obtained after oral administration of* A. japonica* extracts.

The lower limit of quantification (LLOQ) was calculated by the signal-to-noise ratio (S/N) higher than 5 through analyzing the 6 standards spiked in plasma samples. Validation of the method was done in accordance with the guidelines set by the United States Food and Drug Administration (USFDA) [14].

#### 2.6.2. Linearity

An eight-point standard calibration curve was constructed by using the peak area ratio of 6 analysis compounds to the IS against concentration. Each calibration curve was performed individually by using least square weighted (1/*x*) linear regression. The calibration curve was applied to evaluated intraday (on the same day), interday (on three consecutive days), and stability accuracies and precision of the assay.

#### 2.6.3. Precision and Accuracy

Testing of the precision and accuracy was performed using the six batches of QC samples at four different concentrations during 1 day for intraday and within 3 different days for interday. The accuracy and precision were calculated and expressed as the percentage of the measured concentration to the nominal concentration and relative standard deviation (RSD), respectively. The range of the testing should be 85–115% for the accuracy, and the RSD at each concentration level should not exceed 15% of the coefficient of variation (CV) except for the LLOQ, where it should not exceed 20% of the CV.

#### 2.6.4. Recovery and Matrix Effect

Determination of the recovery was carried on through comparing the peak areas of 6 compounds extracted spiked samples with those of the analytes and IS without extraction at correspondent concentrations. The matrix effect was investigated by analytes in postextracted spiked samples with those of the analytes in unextracted samples, at the same concentrations at QC concentrations. The mean value of the recovery and matrix effect should be in the range of 85–115% at three levels of QC sample except for the LLOQ, where it should be in the range of 80–120%.

#### 2.6.5. Stability

The stability for 6 analysis compounds including 24 h stability (keeping the sample in autosampler conditions of room temperature for 24 h), three freeze/thaw cycles (subjected to 3 cycles of freezing at about −20°C and thawing at room temperature), and a long-term stability (stored at about −80°C for 4 weeks) of analytes in plasma was performed by analyzing QC samples at four different concentrations.

### 2.7. Pharmacokinetic Study

Ten male Sprague-Dawley rats (270–280 g) were kept at the animal center of Tianjin University of Traditional Chinese Medicine (Tianjin, China) under environmentally controlled conditions. The room temperature of the animal center was maintained at 25°C and the rats were allowed free access to food and water although only water was offered to rats 12 h prior to the experiment. According to the clinical dose used in human, the rat dose of* A. japonicae* extracts used was 0.93 g kg^−1^. After oral administration of the extract to rat, 200 *μ*L of blood samples was obtained into heparinized 1.5 mL polythene tubes at 5, 10, 15, 30, and 45 min and 1, 2, 4, 6, 8, 12, and 24 h. After blood sample collection into the heparinized 1.5 mL polythene tubes and centrifugation at 6000 rpm for 10 min, the plasma was transferred into clean tubes and stored in a freezer at −20°C until analysis. The pharmacokinetic parameters were determined using pharmacokinetics program (DAS1.0 Medical College of Wannan, China) and performing a one compartmental analysis.

## 3. Results and Discussion

### 3.1. LC-MS/MS Optimization

The MS intensities for the 6 compounds and 3 ISs were optimized following infusion of standard solution of each into the mass spectrometer. The signal intensities of the compounds were found to be greater in the negative modes after optimizing each compound in both positive and negative modes. Deprotonated [M−H]^−^ parent ions were produced from the full scan Q1 mass spectrum for the 6 compounds and 3 ISs at* m/z* 168.8, 327.0, 288.9, 441.1, 462.9, 447.0, 192.8, 415.0, and 307.0 for gallic acid, bergenin, epicatechin, epicatechin gallate, isoquercitrin, and quercetin-3-rhamnoside, ferulic acid (IS1), puerarin (IS2), and warfarin (IS3), respectively. The MS parameters were optimized such as the DP and CE to ensure that MRM transitions were sensitive. The various MRM parameters are listed in Table 1. IS1 and IS3 were used to determine gallic acid and bergenin, respectively. IS2 was selected to determine the four components including epicatechin, epicatechin gallate, isoquercitrin, and quercetin-3-rhamnoside.

**Figure 2:**
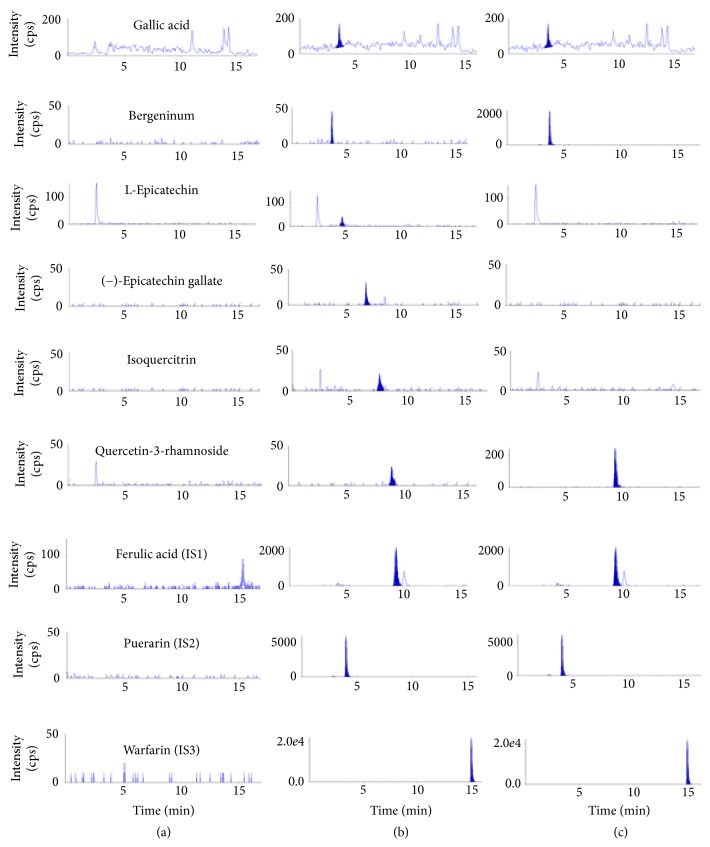
Typical chromatograms of (a) blank rat plasma, (b) blank rat plasma spiked with standard compounds at LLOQs, and (c) real sample from rats at 30 min after oral administration of* A. japonica* extract.

Various mobile phases consisting of different solutions of acetonitrile, methanol, and formic acid were optimized in order to obtain short run times, well-resolved peaks, and peaks with symmetrical shape. In the end, better response, good separation, and increased sensitivity of the compound peaks were obtained using a mobile phase consisting of acetonitrile and 0.05% aqueous formic acid solution. Also, no interference was observed between the tested compounds and internal standards. Typical chromatograms of blank plasma, plasma spiked with the compounds and IS, and plasma with the IS are shown in Figure 2.

### 3.2. Method Validation

#### 3.2.1. Specificity

The method's specificity was evaluated by looking at the MRM chromatograms of blank plasma sample, blank plasma spiked with reference standards, and IS and then plasma sample after giving* A. japonica* extract orally to rats as shown in Figure 2. A good separation of the 6 compounds was achieved without interference peaks from endogenous substances at retention times of gallic acid (4.2 min), bergenin (4.6 min), epicatechin (4.9 min), (−)-epicatechin gallate (7.2 min), isoquercitrin (8.4 min), and quercetin-3-rhamnoside (9.5 min).

#### 3.2.2. Linearity and Sensitivity

The calibration curves for the 6 compounds were linear within the stipulated ranges as shown in Table 2. This was achieved with the help of weighted least square linear regression analysis with a weight factor of 1/*x*. Representative mean calibration curves are as follows: *Y* = 0.0157*X* − 0.0112, *Y* = 0.00125*X* + 0.000782, *Y* = 0.00223*X* + 0.00102, *Y* = 0.00579*X* − 0.00933, *Y* = 0.00582*X* + 0.00202, and *Y* = 0.0156*X* − 0.000007 for gallic acid, bergenin, epicatechin, epicatechin gallate, isoquercitrin, and quercetin-3-rhamnoside, respectively, where *y* represented the peak area ratio of analytes to IS and *x* denoted the concentration of analytes present in the plasma samples. Also, the LLOQs of the analytes are 2, 2, 4, 2, 1, and 0.4 ng mL^−1^ for gallic acid, bergenin, epicatechin, epicatechin gallate, isoquercitrin, and quercetin-3-rhamnoside, respectively. LLOQs of these analytes were sufficiently applied for the pharmacokinetic study in rats.

#### 3.2.3. Precision and Accuracy

The intraday and interday variabilities of the LLOQ and QC samples are shown in Table 3. The intraday and interday precision ranged from 3.87 to 15.7% and 7.31 to 16.2%, respectively, while data on the accuracy was in the range of 85.5 to 114%. All assay values were within stipulated limits and hence suggestive of a reliable, reproducible, and accurate method.

#### 3.2.4. Extraction Recovery and Matrix Effect

As shown in Table 4, the extraction recovery of the analytes was in the range of 89.6% to 111% at three concentration levels. On the other hand, the matrix effects of the six compounds at three concentration levels ranged from 88.5% to 116%. No significant matrix effects on analytes and IS were observed. This indicates that there are no coeluting substances influencing the ionization of the analytes and IS and the good protein precipitation efficiency.

#### 3.2.5. Stability

The stabilities of all analytes were tested through the analysis of the three levels of QC samples as shown in Table 5. It was found that the analytes were considerably stable in rat plasma and processed samples under different storage conditions.

### 3.3. The Content of 6 Compounds in* A. japonica* Extract

The six compounds were determined in* A. japonica *extract. The content of gallic acid, bergenin, epicatechin, epicatechin gallate, isoquercitrin, and quercetin-3-rhamnoside are 2.1467 *μ*g/mg, 24.9 *μ*g/mg, 0.696 *μ*g mg^−1^, 0.198 *μ*g mg^−1^, 0.0916 *μ*g mg^−1^, and 2.13 *μ*g mg^−1^, respectively. Therefore, the administration dosages of gallic acid, bergenin, epicatechin, epicatechin gallate, isoquercitrin, and quercetin-3-rhamnoside after administration of* A. japonica* extract were 2.00 mg kg^−1^, 23.16 mg kg^−1^, 0.65 mg kg^−1^, 0.18 mg kg^−1^, 0.09 mg kg^−1^, and 1.98 mg kg^−1^, respectively.

### 3.4. Application

**Figure 3:**
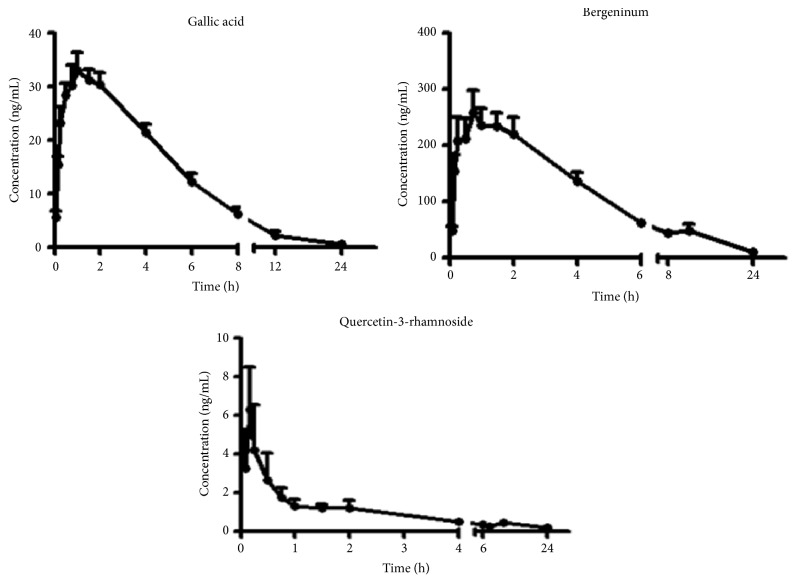
Mean plasma concentration-time profiles of gallic acid, bergenin, and quercetin-3-rhamnoside in rats after oral administration of* A. japonica* extract (*n* = 10, mean ± SD).

The method having been validated was successfully applied to the determination of plasma concentration of 3 of the compounds in rats, after oral administration of* A. japonica* extract at a dose of 0.93 g kg^−1^ to rats. The 3 compounds including gallic acid, bergenin, and quercetin-3-rhamnoside were determined and their plasma concentration-time profiles are illustrated in Figure 3. Their one compartment model pharmacokinetic parameters are shown in Table 6. Gallic acid reached a peak plasma concentration of 35.5 ± 10.2 ng mL^−1^ and the* T*max was 1.40 ± 1.13 h after oral administration of the* A. japonica *extract. The area under the curve (AUC_0–24 h_) and the half-life, *T*_1/2_, were 194 ± 44 ng (mL h)^−1^ and 3.21 ± 4.57 h, respectively. The *C*max and* T*max for bergenin and quercetin-3-rhamnoside were 288 ± 107 ng mL^−1^, 0.86 ± 0.52 h and 7.08 ± 8.28 ng mL^−1^ and 0.50 ± 0.60 h with the area under the curve for each compound being 1743 ± 666 ng (mL h)^−1^ and 6.44 ± 11.99 ng (mL h)^−1^, respectively. Both compounds exhibited a* T*max less than one hour implying that they had a fast rate of absorption into plasma from the gastrointestinal tract after oral administration, compared to gallic acid which achieved maximum plasma concentrations after one hour.

## 4. Conclusions

A sensitive and simple LC-MS/MS method for simultaneous measurement of 6 compounds in rat plasma was developed and subsequently validated. The method proved to be accurate, precise, and specific and was successfully applied to investigate the pharmacokinetics of gallic acid, bergenin, and quercetin-3-rhamnoside after an oral administration of* A. japonica *extracts to rats.

## Figures and Tables

**Table 1:** MRM parameters of six compounds and three ISs.

Compounds	Q1	Q3	DP (V)	EP (V)	CE (V)	CXP (V)
Gallic acid	168.8	124.9	−28	−9	−20	−5
Bergeninum	327.0	191.9	−48	−8	−38	−5
Epicatechin	288.9	108.9	−45	−4.5	−35	−4
Epicatechin gallate	441.1	169.2	−55	−8	−28	−3.5
Isoquercitrin	462.9	300.0	−80	−8	−38	−6.5
Quercetin-3-rhamnoside	447.0	300.0	−60	−4.5	−35	−8
Ferulic acid (IS1)	192.8	134.1	−28	−9	−24	−5
Puerarin (IS2)	415.0	266.9	−62	−8	−45	−6.5
Warfarin (IS3)	307.0	161.1	−41	−9	−27	−7

**Table 2:** The calibration curves, linearity range, and LLOQs of 6 compounds (*n* = 6).

Compounds	Regression equation	*R*	Linearity range	LLOQ (ng ml^−1^)
Gallic acid	*Y* = 0.0157*X* − 0.0112	0.9920	2–500	2
Bergeninum	*Y* = 0.00125*X* + 0.000782	0.9983	2–500	2
Epicatechin	*Y* = 0.00223*X* + 0.00102	0.9984	4–1000	4
Epicatechin gallate	*Y* = 0.00579*X* − 0.00933	0.9940	2–500	2
Isoquercitrin	*Y* = 0.00582*X* + 0.00202	0.9964	1–250	1
Quercetin-3-rhamnoside	*Y* = 0.0156*X* − 0.000007	0.9971	0.4–100	0.4

**Table 3:** Intraday, interday accuracy, and precision of 6 compounds (*n* = 6).

Compounds	Concentration (ng/mL)	Intraday		Interday	
		Accuracy (%)	RSD (%)	Accuracy (%)	RSD (%)
Gallic acid	2	99.4	6.59	96.0	9.67
	6	93.8	9.14	101	9.12
	20	85.5	4.33	86.1	7.57
	200	104	3.87	108	7.31

Bergeninum	2	106	9.08	103	16.2
	6	100	10.7	99	10.4
	20	114	6.40	108	11.4
	200	109	13.4	111	13.2

L-Epicatechin	4	92.8	5.07	96.5	12.2
	12	97.3	8.15	88.9	12.9
	40	94.9	9.63	96.3	12.8
	400	90.0	15.7	94.8	9.62

(−)-Epicatechin gallate	2	93.4	16.0	108	9.97
	6	93.9	7.11	94.6	15.8
	20	86.6	12.2	90.0	14.3
	200	103	10.7	98.9	13.8

Isoquercitrin	1	110	9.45	104	12.4
	3	90.3	7.54	92.4	13.4
	10	101	11.8	101	8.50
	100	109	4.59	102	8.10

Quercetin-3-rhamnoside	0.4	89.1	14.9	91.4	15.6
	1.2	85.6	4.42	87.9	11.8
	4	95.7	14.7	90.1	12.5
	40	99.9	5.01	95.0	8.05

**Table 4:** The recoveries and matrix effects of 6 compounds (*n* = 6).

Compounds	Concentration (ng/mL)	Recovery		Matrix effect	
		Accuracy (%)	RSD (%)	Accuracy (%)	RSD (%)
Gallic acid	2	111	10.2	116	8.89
	6	111	6.76	113	3.52
	20	104	7.92	110	5.03
	200	106	8.50	99.7	9.08

Bergeninum	2	109	8.59	109	2.31
	6	108	12.6	112	14.2
	20	95.4	7.14	107	6.05
	200	91.0	10.2	115	9.46

L-Epicatechin	4	111	13.7	99.5	8.46
	12	105	11.3	102	11.6
	40	110	11.1	88.5	5.87
	400	92.4	6.49	92.0	6.95

(−)-Epicatechin gallate	2	109	6.58	113	10.4
	6	108	9.99	114	5.67
	20	109	9.76	108	7.24
	200	101	5.08	115	13.5

Isoquercitrin	1	103	10.2	112	1.72
	3	90.0	12.3	106	13.3
	10	107	8.03	113	8.65
	100	98.3	5.63	113	6.28

Quercetin-3-rhamnoside	0.4	104	10.9	110	10.1
	1.2	108	13.6	110	11.8
	4	89.6	3.14	110	11.9
	40	97.9	3.59	118	6.77

Ferulic acid	100	80.0	2.76	87.7	3.10

Puerarin	100	85.7	6.55	79.4	2.65

Warfarin	100	76.4	3.77	106	3.41

**Table 5:** Stability of the 6 compounds (*n* = 6).

Compounds	Concentration (ng/mL)	24 h stability		Free-throw 3 times		Long stability (1 month)	
		Accuracy (%)	RSD (%)	Accuracy (%)	RSD (%)	Accuracy (%)	RSD (%)
Gallic acid	2	116	8.59	92.5	9.19	95.6	11.4
	6	111	7.23	114	8.03	101	7.64
	20	85.8	3.54	94.0	7.77	98.4	5.65
	200	103	4.32	101	10.6	92.7	7.51

Bergeninum	2	83.4	14.4	106	5.81	82.3	11.4
	6	105	9.58	104	12.3	91.3	16.4
	20	92.5	13.3	94.0	4.95	112	9.97
	200	106	8.45	87.3	10.9	114	7.59

Epicatechin	4	91.8	4.22	105	12.6	114	6.65
	12	95.1	10.6	111	7.78	104	7.25
	40	96.6	9.33	111	7.27	113	9.54
	400	97.6	4.69	99.4	2.79	102	4.47

Epicatechin gallate	2	108	5.75	109	11.7	87.1	6.45
	6	100	11.9	98.4	7.56	88.9	10.8
	20	85.3	3.72	91.4	3.89	100	7.17
	200	89.7	6.01	88.0	6.78	105	4.88

Isoquercitrin	1	111	6.64	105	13.0	109	11.9
	3	99.1	11.8	103	9.81	100	9.45
	10	96.7	5.57	96.9	3.54	99.9	6.89
	100	92.9	4.54	91.0	6.77	91.3	8.75

Quercetin-3-rhamnoside	0.4	93.7	9.37	115	3.67	114	8.76
	1.2	96.3	7.49	90.4	8.84	92.2	7.77
	4	93.3	9.24	93.3	3.98	90.6	8.70
	40	87.3	5.65	85.0	4.94	85.0	6.51

**Table 6:** The pharmacokinetic parameters of 3 compounds.

Parameters	Gallic acid	Bergeninum	Quercetin-3-rhamnoside
*T*max (h)	1.40 ± 1.13	0.86 ± 0.52	0.50 ± 0.60
*C*max (ng/mL)	35.5 ± 10.2	288 ± 107	7.08 ± 8.28
*T*_1/2_ (h)	3.21 ± 4.56	3.87 ± 1.62	2.59 ± 2.80
AUC_(0–24 h)_ (ng/mL*∗*h)	194.4 ± 44.0	1743 ± 666	6.44 ± 12.00
AUC_(0-∞)_ (ng/mL*∗*h)	199.7 ± 43.9	1841 ± 769	6.95 ± 20.67
MRT_(0–*t*_24 h_)_ (h)	4.50 ± 1.15	6.04 ± 1.05	5.08 ± 6.36
MRT_(0–*∞*)_ (h)	5.33 ± 1.61	7.26 ± 2.17	7.09 ± 19.6
